# The effect of gum chewing on xerostomia and salivary flow rate in elderly and medically compromised subjects: a systematic review and meta-analysis

**DOI:** 10.1186/s12903-023-03084-x

**Published:** 2023-06-20

**Authors:** Michael W. J. Dodds, Mohamed Ben Haddou, Jon E. L. Day

**Affiliations:** 1 Mars Wrigley, 1132 West Blackhawk Street, Chicago, IL 60642 USA; 2 Mentis Sa, Avenue Louise 367, 1050 Brussels, Belgium; 3 Cerebrus Associates, The White House, 2 Meadrow, Godalming, GU7 3HN Surrey UK

**Keywords:** Xerostomia, Dry mouth, Chewing gum, Mastication, Systematic review, Meta-analysis, Salivary flow rate

## Abstract

**Background:**

Xerostomia negatively affects quality of life. Symptoms include oral dryness; thirst; difficulty speaking, chewing, and swallowing food; oral discomfort; mouth soft tissue soreness and infections; and rampant tooth decay. The objective of this systematic review and meta-analysis was to investigate if gum chewing is an intervention that results in objective improvements in salivary flow rates and subjective relief from xerostomia.

**Method:**

We searched electronic databases including Medline, Scopus, Web of Science, Embase, Cochrane Library (CDSR and Central), Google Scholar and the citations of review papers (last searched 31/03/23). The study populations included: 1) elderly people with xerostomia (> 60 years old, any gender, and severity of xerostomia), and 2) medically compromised people with xerostomia. The intervention of interest was gum chewing. Comparisons included gum chewing vs. no gum chewing. The outcomes included salivary flow rate, self-reported xerostomia, and thirst. All settings and study designs were included. We conducted a meta-analysis on studies where measurements of unstimulated whole salivary flow rate for both a gum chewing, and no gum chewing intervention (daily chewing of gum for two weeks or longer) were reported. We assessed risk of bias using Cochrane’s RoB 2 and ROBINS-I tools.

**Results:**

Nine thousand six hundred and two studies were screened and 0.26% (*n* = 25) met the inclusion criteria for the systematic review. Two of the 25 papers had a high overall risk of bias. Of the 25 papers selected for the systematic review, six met the criteria to be included in the meta-analysis which confirmed a significant overall effect of gum on saliva flow outcomes compared to control (SMD = 0.44, 95% CI: 0.22—0.66; *p* = 0.00008; I^2^ = 46.53%).

**Conclusions:**

Chewing gum can increase unstimulated salivary flow rate in elderly and medically compromised people with xerostomia. Increasing the number of days over which gum is chewed increases the improvement in the rate of salivation. Gum chewing is linked with improvements in self-reported levels of xerostomia (although it is noted that no significant effects were detected in five of the studies reviewed). Future studies should eliminate sources of bias, standardise methods to measure salivary flow rate, and use a common instrument to measure subjective relief from xerostomia.

**Study registration:**

PROSPERO CRD42021254485.

## Background

It has been estimated that up to 39% of the non-institutionalised older adult population suffer from xerostomia [1], with a reported overall prevalence of 21.3% in males and 27.3% in females across ages 20 – 80 years [2]. Xerostomia is the subjective perception of oral dryness, and is often caused by salivary gland hypofunction resulting in low salivary output. However, it is notable that subjective xerostomia does not always correspond with objective measures of salivary flow rate [3]. The main factors causing decreased saliva generation include natural outcomes of aging, side effects of medication or medical procedures such as head and neck radiation therapy and haemodialysis, and specific medical or psychiatric conditions such as connective tissue disorders, diabetes, anxiety and depression [4].

Xerostomia negatively affects the Oral Health Quality of Life index [5]. Symptoms include sensations of dryness or thirst, difficulty speaking, chewing and swallowing food, oral discomfort, and mouth soft tissue soreness. Xerostomia can also lead to oral infection and increased incidence of dental caries [2]. One approach to mitigate these symptoms is the use of sugar-free chewing gum which stimulates a strong flow of saliva through the separate and interactive effects of mastication and taste [6]. It has been used to provide symptomatic relief in patients suffering from xerostomia or salivary gland hypofunction. A Cochrane Collaboration review of topical therapies for dry mouth concluded that chewing gum increased saliva production in those with residual secretory capacity. Chewing gum may be preferred by patients, however, the review found no evidence to suggest the effect is greater or worse in comparison to saliva substitutes [7]. A more recent integrative review concluded that chewing gum for treatment of thirst resulted in increased salivary flow, xerostomia relief, and thirst reduction [8]. However, neither of these reviews included a meta-analysis of salivary flow rate data. Therefore, the objective of our systematic review and meta-analysis was to determine whether gum chewing leads to salivation and consequent relief from xerostomia in elderly and medically compromised people. Such evidence could support the development of interventions that address xerostomia and improve quality of life in both healthy and challenged populations.

## Methods

### Protocol registration

The protocol was registered with the international prospective register of systematic reviews (PROSPERO) in accordance with PRISMA-P guidelines (PROSPERO CRD42021254485). The protocol can be accessed at: https://www.crd.york.ac.uk/prospero/display_record.php?RecordID=254485.

### Defining the research question

We formulated the research question of the systematic review using PICOS (Population, Intervention, Comparison, Outcome, and Setting) [9]. The populations studied were: 1) elderly people with xerostomia (> 60 years old, any gender, and severity of xerostomia), and 2) medically compromised people with xerostomia. These populations were not restricted to any geography and included papers from all over the world. The intervention of interest was gum chewing (with or without specific ingredients designed to promote salivation). The comparisons included gum chewing vs. no gum chewing. The outcomes used for study selection included rate of salivation / salivary volume per unit time, xerostomia relief (self-reported, e.g., the Xerostomia Inventory), and thirst. Settings of any type (e.g. laboratory, clinical, nursing home, etc.), study designs of any type (e.g. RCTs including within- and between-subjects’ designs, cross-sectional, etc.) were in scope. Only fully-refereed research studies published in the English language were included. Abstracts and review papers were excluded.

### Data sources and searches

We searched the Medline (1946 to 31^st^ March 2023) and Scopus (1806 to 31^st^ March 2023), Web of Science, Embase and Cochrane Library (CDSR and Central) databases using the syntax outlined in Table 1. Additionally, we searched for randomised controlled trials in clinical trials registries (https://clinicaltrials.gov/ and https://www.isrctn.com/), with terms relating to xerostomia, dry mouth, chewing, mastication, gum, salivation, oral hydration, and thirst. Other methods used for identifying relevant research included laddering (manual searching) from references cited in the literature obtained, identifying possible data from conferences attended, and reviewing proprietary information.

**Table 1:** The search syntax used to return 9,602 records in MEDLINE, Scopus, Web of Science and Cochrane Library (CDSR and Central)

Stage	Syntax
1	Medline: ((chew* or mastication or gum or paraffin) not (mouse or mice or rodent* or dairy or cow or cat or chicken or pig or donkey or mule or grazing or tobacco or smoking or khat or wine)).ab,kw,ti AND ((xerostomia or dry mouth or saliva* or thirst) not (mouse or mice or rodent* or dairy or cow or cat or chicken or pig or donkey or mule or grazing or tobacco or smoking or khat or wine)).ab,kw,ti
2	Scopus: TITLE-ABS-KEY ( ( ( chew* OR mastication OR gum OR paraffin) AND NOT ( mouse OR mice OR rodent* OR dairy OR cow OR cat OR chicken OR pig OR donkey OR mule OR grazing OR tobacco OR smoking OR khat OR wine))) AND ( LIMIT-TO ( SRCTYPE, "j")) AND ( LIMIT-TO ( LANGUAGE, "english")) AND TITLE-ABS-KEY ( ( ( xerostomia OR dry AND mouth OR saliva* OR thirst) AND NOT ( mouse OR mice OR rodent* OR dairy OR cow OR cat OR chicken OR pig OR donkey OR mule OR grazing OR tobacco OR smoking OR khat OR wine))) AND ( LIMIT-TO ( SRCTYPE, "j")) AND ( LIMIT-TO ( LANGUAGE, "english"))
3	Web of science: (chew* OR mastication OR gum OR paraffin) and (xerostomia OR dry AND mouth OR saliva* OR thirst)
4	CDSR: (chew* OR mastication OR gum OR paraffin) and (xerostomia OR dry AND mouth OR saliva* OR thirst)
5	Central: (chew* OR mastication OR gum OR paraffin) and (xerostomia OR dry AND mouth OR saliva* OR thirst)
6	1 and 2 and 3 and 4 and 5
7	Deduplicate 6

### Study selection

The papers identified by the searches were independently reviewed by two researchers (JD & MD) to assess them against the inclusion and exclusion criteria of the PICOS statement. A consensus meeting was held to discuss papers where there was disagreement on whether they should, or should not, be included.

### Outcomes and variables

For the systematic review, the outcome measures were self-reported xerostomia, salivary flow rates (stimulated and unstimulated), and thirst. For the meta-analysis, the primary outcome measure was unstimulated salivary flow rate (ml / min). Since the immediate effect of chewing sugar-free gum is to increase saliva flow, we determined that unstimulated saliva flow rate would be the preferred outcome measure for the meta-analysis, since this would be more consistent across studies, and would be least influenced by different stimuli used to collect saliva. Furthermore, unstimulated, but not stimulated, salivary flow rate has been found to be significantly correlated with xerostomia symptoms [10].

### Data extraction and quality assessment

Following the selection of papers, the two reviewers independently examined each paper for risk of bias using Cochrane’s RoB 2 (for individually-randomised, parallel-group trials and cross-over trials) [11] and ROBINS-I tools [12]. A second consensus meeting was held to decide on the final, agreed, risk of bias.

In addition to the systematic review, a meta-analysis was conducted on a subset of the selected papers where both a gum chewing intervention was imposed for two or more weeks, and where unstimulated salivary flow rate was measured (ml / min). Data extraction was performed independently by two reviewers (JD & MBH) and the results compared to highlight any potential errors. Data were extracted from the text, tables and figures of each of the papers. Additionally, we also recorded: 1) authors and publication year, 2) sample size (control and intervention), 3) intervention protocol (observation weeks), and 4) outcome data expressed as saliva flow rate. Where the data were reported in graphical form, WebPlotDigitizer [13] was used to extract the underlying numerical data. Where repeated measures were reported (i.e. multiple observations over a number of weeks), only the outcomes where the intervention had been imposed for two or more weeks were included.

### Data synthesis and analysis

A random-effects meta-analysis using standardised mean differences (SMD) was performed using RStudio software (R version 4.1.3 (2022–03-10)) to assess the overall effect of gum chewing on unstimulated salivary flow rate. The data were converted to SMDs (Hedge’s g) and standard errors to obtain 95% confidence intervals (CIs). The following data were used for the calculation of SMDs: 1) mean ± standard deviation, and 2) sample size (n). None of the included studies reported the correlations between trials, thus a 0.5 correlation was assumed for all trials, as per recommendations [14]. Hedge’s g values of < 0.2, 0.2 ≤ 0.5, 0.5 – 0.8, and > 0.8 were considered to represent very small, small, medium, and large effects respectively [15]. The I^2^ statistic was used to assess the degree of heterogeneity, with values from < 50% indicating low heterogeneity, 50–75% moderate heterogeneity, and > 75% high level of heterogeneity. Heterogeneity between the studies was assessed using graphic exploration with funnel plots.

To assess the stability of the results and investigate the effects of outliers, a “leave one out” approach was conducted. In this approach, studies are removed one by one, and the random effect model is fitted on the remaining studies. Meta-regression was used to investigate whether the duration of the intervention (e.g. how many days participants chewed gum) influenced unstimulated salivary flow rates.

## Results

### Search results

**Fig. 1:**
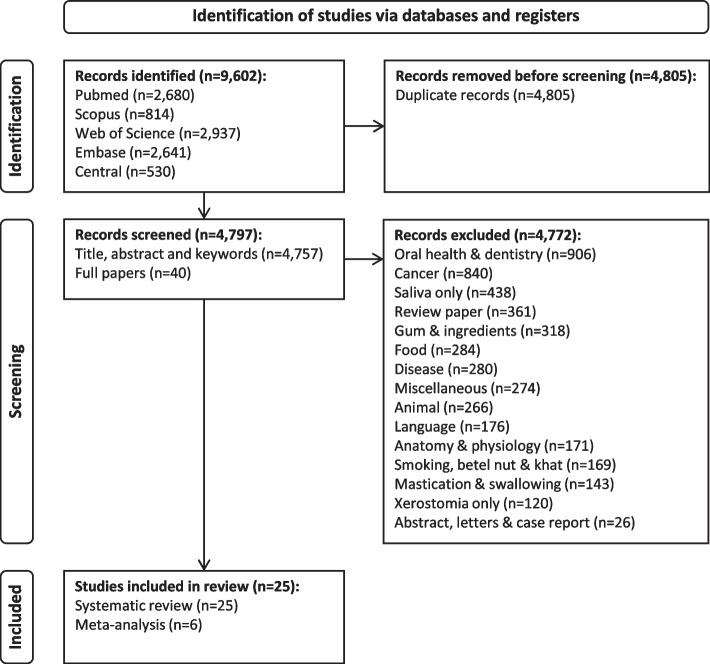
Overview of the systematic review on the effect of gum chewing on xerostomia and salivary flow rate in elderly and medically compromised subjects (as per PRISMA statement)

The search returned a list of 9,602 papers. A summary of the number and types of exclusions is given in Fig. 1. The two reviewers disagreed over the classification of 40/9,602 papers in their independent reviews (0.76%). A consensus meeting was used to decide on the ultimate inclusion and exclusion of the disputed papers resulting in a final list of 25 publications for the systematic review [16–40].

### Characteristics of included studies

Each paper was reviewed to determine the experimental design, the numbers of participants, the outcome measures, and the key results (Table 2).

**Table 2:** Characteristics of the studies included in the systematic review of studies addressing the relationship between mastication, salivation and xerostomia

Reference	Participants	Intervention			Results
		**Design**	**Intervention**	**Outcome measures**	
Allida et al. (2021) [16]	71 patients with chronic heart failure	A prospective, randomised, open label, design with two treatments: 1) chewing gum (*n* = 36), and 2) no chewing gum (*n* = 35)	Participants instructed to chew the gum gently, for at least 10 min, six times a day and as desired throughout the day when the mouth felt dry or when they were thirsty. The intervention period was two weeks	**Thirst:** Measured in the short-term (Days 1–4 days) and longer-term (at Days 7, 14 & 28) using a Visual Analogue Scale (VAS), and a numeric rating scale (NRS) for the questions: 1) ‘How is your thirst now?’, 2) ‘How has your thirst been in the last 24 h on average?’ and 3) ‘What is the worst your thirst has been in the last 24 h?’	**Short-term thirst:** (Day 4): Chewing gum significantly ↓ levels of thirst compared to control on Day 4 (*) and Day 14 (*) **Longer-term thirst:** (Days 7, 14 and 28): Thirst in the last 24 h was ↓ compared to control on Day 7 (** & * for VAS and NRS respectively). Instantaneous thirst was ↓ compared to control on Day 14 (* & * for VAS and NRS respectively). Thirst in the last 24 h was ↓ compared to control on Day 28 (*** & * for VAS and NRS respectively)
Kim et al. (2021) [17]	96 participants aged 65 years or older	A prospective, randomised, design with three treatments: 1) simple oral exercise (SOE, v = 41), 2) simple oral exercise + chewing gum group (GOE, *n* = 40), and 3) control group (CON, *n* = 21)	SOE comprised lip, tongue, and cheek stretching, as well as a masticatory and swallowing exercises. GOE participants were additionally instructed to chew the gum in a habitual manner twice a day for 10 min. CON participants were not instructed to perform any oral exercises. The intervention period was 8 weeks followed by a 3-week maintenance period	**Saliva flow rate:** Unstimulated saliva was collected by asking participants to expectorate saliva into a test tube once every minute for 5 min	**Saliva flow rate**: SOE significantly↑ unstimulated salivary flow rate compared with baseline during the intervention period (***). At 8 weeks, mean differences in unstimulated saliva flow rate with baseline were 3.6-times ↑ for SOE and 2.2 times ↑for GOE compared with CON (*)
				**Symptoms of dry mouth:** Self-reported discomfort during mastication, aspiration during swallowing, and symptoms of dry mouth were assessed using a questionnaire (nominal scale after Torres et al., 2002 and Fox et al., 1987)	**Symptoms of dry mouth:** No significant differences in self-reported measures were detected (NS)
Ozen et al. (2021) [18]	44 patients undergoing haemodialysis	A prospective randomised, controlled, single-blind, study with two treatments: 1) chewing gum (*n* = 22), and 2) no chewing gum (*n* = 22)	Commercially available gum was chewed for 10 min six times a day, and when the patients felt mouth dryness or were thirsty. The intervention period was 3 months	**Xerostomia:** Measured using Visual Analogue Scale (VAS)	**Xerostomia**: Chewing gum significantly ↓ levels of xerostomia compared to control in the 2^nd^ (**) and 3^rd^ month (***)
				**Saliva flow rate:** Measured at the first, 12^th^, and 36^th^ haemodialysis session at baseline, week 4, and week 12	**Saliva flow rate:** Chewing gum significantly ↑ salivary flow rate compared to control in the 3^rd^ month (***)
Kaee et al. (2020) [19]	91 participants, more than six months following radiotherapy for head and neck cancer	A prospective, non-blinded, randomised, design with two treatments: 1) daily chewing gum (*n* = 55), and 2) standard care (*n* = 36)	Sugar-free gum was chewed five times daily for at least five minutes. Standard care included aid with water, saliva substitutes or stimulants already part of daily routines. The intervention period was 4 weeks	**Xerostomia-related QOL:** Measured using both the single-item ‘Dry Mouth’ in the EORTC QLQ-H&N35 questionnaire, and the Groningen Radiation-Induced Xerostomia (GRIX) questionnaire	**Xerostomia**: Chewing gum was associated with a significant ↓ in dry mouth (EORTC QLQ-H&N35) (**), and a significant ↓ in xerostomia during the day (EORTC QLQ-H&N35) (***)
				**Rate of salivation** Unstimulated and stimulated saliva was collected by asking participants to expectorate saliva into a test tube for 5 min	**Rate of salivation**: Salivary flow ↑ following 5 min of stimulation within both arms (*** respectively). However no significant differences were detected between arms (NS)
Garcia et al. (2019) [20]	102 patients with thirst intensity greater than or equal to three (as assessed by the Numeral Verbal Scale)	A randomised controlled clinical trial, with parallel treatments: 1) menthol chewing gum (*n* = 51), and 2) control group (*n* = 51)	TRIDENT mentholated gum was chewed for 10 min in a natural rhythm. The intervention period was the preoperative period	**Thirst intensity:** Measured using the Numeral Verbal Scale	**Thirst intensity:** Chewing significantly ↓ the variation in thirst intensity (***)
				**Thirst discomfort:** Measured using the Perioperative Thirst Discomfort Scale	**Thirst discomfort:** Chewing significantly ↓ the variation in thirst intensity (***)
Dehghanmehr et al. (2018) [21]	50 patients undergoing haemodialysis	A quasi-experimental trial with two treatments: 1) chewing gum (*n* = 25) and 2) no chewing gum (*n* = 25)	Patients of intervention group were instructed to chew sugar free gum for one week when they felt thirsty. The control group did not receive any intervention	**Thirst:** Measured using the dialysis thirst quotient instrument (DTI, dry mouth measurement tools (XI), VAS)	**Thirst:** Chewing gum significantly ↓ thirst over the intervention period (*) whereas no significant changes were detected in the control group (NS). Thirst was significantly ↓ in the chewing gum group compared with the control group post-intervention (*)
				**Dry mouth:** Measured using the xerostomia inventory (XI)	**Dry mouth:** Chewing gum significantly ↓ dry mouth over the intervention period (***) whereas no significant changes were detected in the control group (NS). Dry mouth was signicantly ↓ in the chewing gum group compared with the control group post-intervention (***)
Nakagawa et al. (2017) [22]	12 participants with a mean age of 77.8 ± 4.6 years	A within subjects design with two gum chewing exercise treatments: 1) with soft gum (GCE-S, *n* = 12) and 2) with hard gum (GCE-H, *n* = 35)	Participants chewed the soft gum twice daily for 5 min over a 2-week period. There was a 2-week wash-out period, and then participants chewed the hard gum twice daily for 5 min over a 2-week period. In both cases, the participants were instructed to chew at the defined rhythm for the first 2 min and then chew freely for the remaining 3 min	**Mucosal moisture:** Measured on the tongue surface using an oral moisture checking device	**Mucosal moisture:** No significant differences in mucosal moisture were detected before and after GCE-S (NS). Mucosal moisture level of after GCE-H was significantly ↑ than that of before GCE-S (*) and before GCE-H (*)
				**Saliva flow rate:** Measured using a cotton swab which was placed on to floor of the mouth for 30 s and weighed before and after	**Saliva flow rate:** Resting saliva secretion after GCE-S was significantly ↑than that before GCE-S (*). After a 2-week rest period, however, resting saliva secretion before GCE-H was significantly lower than after GCE-S (*). Resting saliva secretion after GCE-H was significantly ↑than before GCE-S (*) and before GCE-H (*)
Duruk & Eser (2016) [23]	61 patients undergoing haemodialysis	A randomised, controlled, single-blind, crossover study with two treatments: 1) gum chewing (*n* = 61), and no gum chewing (*n* = 61)	Participants chewed gum hourly for 15 min in hours 1–4 of dialysis). The gum was Vivident Xylitol sugar-free sweetened gum. The intervention period was a 4-h dialysis session. There was a 15-day wash-out between sessions	**Xerostomia:** Measured using a form for assessing the symptoms of dry mouth (11 × VAS)	**Xerostomia:** On days where gum was chewed, dry mouth symptom scores significantly ↓ between 0 and 4 h for all dimensions in both treatments (all ***), except for the scores for the ‘‘amount of saliva’’ and ‘‘burning sensation in the mouth’’. Similarly, on days where gum was not chewed, dry mouth symptoms scores significantly ↓ between 0 and 4 h for all dimensions in both treatments (* to ***), except for the scores for the ‘‘amount of saliva’’ and ‘‘burning sensation in the mouth’
				**Saliva flow rate:** Saliva was collected at the 0, 1, 2, 3 and 4 h time points by spitting into a tube	**Saliva flow rate:** In both treatments salivary flow rates ↑ between the 0 h and 4 h measurements, however, no significant treatment differences were detected (NS). On the day when gum was not chewed, no significant differences in salivary flow rates were detected during the first hour (NS). On the day when gum was chewed, salivary flow rates ↑ significantly during the first, second, third, and fourth hours when compared with the 0-h time point (*)
Gueimonde et al. (2016) [24]	54 adult volunteers with hypo-salivation	A double blind, randomised, placebo controlled design with three parallel treatments: a) a placebo chewing gum (*n* = 19), b) gum containing Bifidobacterium animalis spp lactis (*n* = 17), and c) gum containing Lactobacillus rhamnosus plus Bifidobacterium longum 46 and Bifidobacterium longum 2C (n = 18)	Participants chewed one gum pastille for 30 min twice daily, in the morning and the evening. The intervention period was 12 weeks	**Saliva flow rate:** Unstimulated saliva was collected using the drainage technique where saliva was allowed to flow into a sterile container for 5 min after which the accumulated saliva was spat into the container. Stimulated saliva was collected using the same method following administration of fresh lemon to the major salivary glands	**Saliva flow rate:** No statistically significant differences were detected between treatments (NS). Chewing gum (with or without probiotics) significantly ↑ unstimulated saliva flow rate (*)
				**Symptoms of dry mouth:** Self-reported symptoms related to dry mouth on a scale of 0 to 4	**Symptoms of dry mouth:** Chewing gum (with or without probiotics) ↓ self-reported dry mouth sensation
Kaae et al. (2016) [25]	31 patients treated for oral or oropharyngeal cancer with slight, moderate, or severe xerostomia	Non-randomised cohort study	Participants chewed a tasteless sugar-free chewing gum for 5 min supported by a metronome (60 beats/min). The study chewing gum was distributed for use at home in-between visits, and patients were instructed to use it three to five times a day including before regular meals. The intervention period was two weeks	**Saliva flow rate:** Unstimulated saliva was collected for 5-min using a spitting method. Stimulated saliva was collected for 5-min following the chewing gum intervention	**Saliva flow rate:** Before and after the intervention period, chewing gum significantly ↑ saliva flow rate. However, no significant difference unstimulated saliva flow rate was detected across the intervention period
				**Xerostomia:** Oral well-being was estimated using the abbreviated EORTC H&N35 questionnaire	**Xerostomia:** Chewing gum significantly ↑ the subjective feeling of total amount of saliva in the mouth (**). In a total of 19 patients, 95% reported a subjective increase in saliva flow after intervention with the chewing gum
Fan et al. (2013) [26]	11 patients undergoing haemodialysis	Cross-over design with two treatments: a) chewing gum (*n* = 11), and b) chewing a plastic straw (*n* = 11)	The participants were instructed to chew one piece of gum gently, for at least 10 min, six times a day and as desired throughout the day when the mouth felt dry or when they were thirsty, from 6–10 pieces approximately. The intervention period was two weeks with a 2-week wash-out between periods	**Thirst:** Assessed by 100-mm visual analogue scales (VAS) and dialysis thirst inventory (DTI)	**Thirst:** Chewing gum significantly ↓ both VAS and DTI measures of thirst (*** and * respectively)
				**Xerostomia:** Assessed by VAS and xerostomia inventory (XI)	**Xerostomia**: Chewing gum significantly ↓ both VAS and XI measures of xerostomia (*** and *** respectively)
				**Saliva flow rate:** Unstimulated whole saliva and paraffin chewing stimulated whole saliva collected for 5 min using a spitting technique	**Saliva flow rate:** No significant differences in saliva flow rates were detected (NS)
Said & Mohammed (2013) [27]	60 patients undergoing dialysis	A quasi-experimental design with two groups: 1) sugar-free chewing gum (*n* = 30), or 2) a control not using gum (*n* = 30)	Participants chewed low- tack, menthol-containing, sugar-free chewing gum for > 10 min, 6 times a day and as desired throughout the day when the mouth felt dry or when they were thirsty	**Xerostomia:** Assessed by VAS and xerostomia inventory (XI)	**Xerostomia:** Over six sessions, chewing gum ↓ xerostomia, whereas xerostomia ↑ in the control group (*** and * respectively)
				**Thirst:** Measured using the dialysis thirst inventory (DTI)	**Thirst:** Over six sessions chewing gum ↓ thirst, whereas thirst ↑in the control group (*** and *** respectively)
				**Salivary flow rate:** Unstimulated and stimulated saliva volumes were measured gravimetrically	**Salivary flow rate:** Over six sessions chewing gum ↑ salivary flow rate, whereas salivary flow rate ↓ in the control group (*** and *** respectively)
Al-Haboubi et al. (2012) [28]	186 participants aged 60 years or older	A single blind, randomised, controlled trial with two treatments: 1) chewing xylitol-containing gum (*n* = 95), or 2) a control group with no gum (*n* = 91)	Participants chewed sugar-free gum twice a day, for 15 min. The control group, on the other hand, were not given chewing gum. The intervention period was 6-months	**Saliva flow rate:** Unstimulated saliva was collected for 5-min using a spitting method. Stimulated saliva was collected for 5-min following chewing a piece of paraffin wax	**Saliva flow rate:** No significant difference in the saliva flow rate of the gum chewing group was detected (NS), whereas the saliva flow of the control group ↑ significantly over the intervention period (***)
Jagodzińska et al. (2011) [29]	38 patients undergoing haemodialysis	A prospective pre/post (3 + 1 month[s]) study	Participants slowly chewed gum, three times each day, after the consumption of the main meals for a minimum of 20 min, and throughout the day only when they were thirsty or perceived xerostomia	**Xerostomia and thirst**: Measured using a questionnaire containing 19 multiple choice questions to assess the effectiveness, preferences, and potential side-effects of the treatments	**Xerostomia and thirst**: No significant differences were detected (NS)
Bots et al. (2005a) [30]	65 patients undergoing haemodialysis	A randomised crossover study with two treatments: 1) sugar-free chewing gum (*n* = 65), and 2) a xanthan gum-based artificial saliva (*n* = 65)	Participants were instructed to chew one or two pieces of gum gently, six times a day for at least 10 min and as desired throughout the day when their mouth felt dry. The intervention period was 6 weeks with a 2-week wash-out period between treatments	**Xerostomia:** Measured using the xerostomia inventory (XI). This is a validated questionnaire with 11 items, each with a 5-point Likert scale. The scores are summed to provide an individual XI score ranging from 11 (no dry mouth) to 55 (extremely dry mouth)	**Xerostomia:** Participants rated chewing gum as significantly ↑ effective at relieving thirst (***) and dry mouth (***) than the artificial saliva
Bots et al. (2005b) [31]	65 patients undergoing haemodialysis	A randomised crossover design with repeated measures and two-treatments: 1) chewing gum (*n* = 65), and 2) a saliva substitute (*n* = 65)	Participants were instructed to chew one or two pieces of gum gently, six times a day for at least 10 min and as desired throughout the day when their mouth felt dry. The intervention period was 2 weeks with a 2-week wash-out period between treatments	**Xerostomia:** Measured using the xerostomia inventory (XI)	**Xerostomia:** Chewing gum significantly ↓ (improved) XI scores from baseline (*)
				**Thirst:** A shortened version of the dialysis thirst inventory (DTI)	**Thirst:** Both chewing gum and saliva substitute significantly ↓ DTI scores (*)
				**Saliva flow rate:** Unstimulated whole saliva and paraffin chewing stimulated whole saliva were both collected before dialysis	**Saliva flow rate:** No significant differences due to treatment were detected (NS)
Simons et al. (2002) [32]	53 participants aged 65 years or older	A randomised, double-blind, placebo-controlled with two treatments: 1) chlorhexidine / xylitol chewing gum (*n* = 26), and 2) xylitol chewing gum (*n* = 27)	Participants chewed two gum pellets for 10 min after breakfast and after the evening meal. The intervention period was 14 days	**Saliva flow rate:** Unstimulated whole saliva and paraffin chewing stimulated whole saliva were both collected	**Saliva flow rate:** No significant differences due to treatment could be detected (NS)
Davies (2000) [33]	43 patients receiving specialist palliative care for advanced cancer	A prospective, randomised, open, crossover study with two treatments: 1) a mucin-based artificial saliva (*n* = 43), and 2) a low-tack, sugar-free chewing gum (*n* = 43)	Participants were instructed to gently chew the gum using both sides of their mouth for at least 10 min before breakfast, before lunch, before dinner and before bedtime and as desired throughout the day when the mouth felt dry. The intervention period was 5 days with a 2 day wash-out period between treatments	**Xerostomia**: Measured using a combination of VAS (Anchor points ‘worst imaginable dryness’ [0 mm] to ‘no dryness [100 mm]) and a questionnaire with scales measuring self-reported mouth dryness and the question ‘Do you think the treatment has helped your dry mouth?’	**Xerostomia**: No significant differences due to treatment were detected (NS)
Stewart et al. (1998) [34]	80 individuals with chronic xerostomia	Randomised, cross-over design with four treatments: 1) a sorbitol/xylitol-sweetened chewing gum (*n* = 80), 2) a sorbitol-sweetened sour lemon lozenge (*n* = 80), and 3) a sorbitol/xylitol-sweetened artificial saliva substitute spray (*n* = 80)	The subjects were instructed to use each product ad libitum for 2 weeks in accordance with the manufacturer’s recommendations. The intervention period was 2 weeks with a 1-week wash-out period between treatment blocks	**Saliva flow rate:** Unstimulated saliva was collected using a “low force spitting” method for 10 min. Baseline stimulated salivary flow rates was determined using the same method with paraffin wax. Post-trial stimulated salivary flow rate was determined with the assigned product as a stimulant	**Saliva flow rate:** No significant differences due to treatment were detected (NS)
				**Xerostomia:** Product preference for relieving symptoms related to hyposalivation was assessed at each post-trial appointment by means of a product rating questionnaire	**Xerostomia:** No significant differences due to treatment were detected (NS)
Simons et al. (1997) [35]	111 participants aged 60 and older	A controlled, double-blind trial with three treatments: 1) chlorhexidine acetate/xylitol gum (ACHX, *n* = 55), 2) xylitol gum (X, *n* = 57), and 3) no-gum control (N, *n* = 52)	Participants in the gum groups chewed two pellets for 15 min twice daily. The intervention period was 12 months	**Saliva flow rate:** Unstimulated whole saliva and paraffin chewing stimulated whole saliva were both collected for a period of 5 min	**Saliva flow rate:** Chewing (both ACHX and X) both significantly ↑ stimulated whole salivary flow rates increased over the 12-month test period (**). No similar effects were detected in the N group (NS)
Risheim & Arneberg (1993) [36]	18 rheumatic patients with dry mouth symptoms and low salivary flow rates	A cross-over design with two treatments: 1) a xylitol sweetened chewing gum (*n* = 18), and 2) a xylitol/sorbitol-sweetened lozenge (*n* = 18)	The subjects were instructed to chew two sticks at a time for 30 min, twice daily from day 1 to day 4, and five times daily from day 5 to day 14. One lozenge was taken four to eight times per day. The intervention period was 2 weeks with a 2-week wash-out period between treatments	**Saliva flow rate:** Resting flow was measured before (PRESTIM) a chewing stimulated flow rate test (STIM), and also 5 min after (POSTSTIM)	**Saliva flow rate:** No significant differences due to treatment were detected in STIM flow (NS). In the lozenge regimen, PRESTIM and POSTSTIM saliva flow rates significantly ↑ increased before and after the intervention (** and ** respectively). In the chewing gum regimen. POSTSTIM saliva flow rates significantly ↑ increased before and after the intervention (*). No similar PRESTIM effects were detected for the chewing gum regimen (NS)
Aagaard et al. (1992) [37]	43 patients complaining of dry mouth	A placebo-controlled double-blind crossover study with three treatments: 1) V6 chewing gum (*n* = 43), 2) an experimental mucin containing chewing gum (*n* = 43), and 3) a placebo chewing gum without flavours (*n* = 43)	Patients were instructed to use the products ad libitum. They were, however, instructed to use the products at least once daily regardless of their opinion regarding taste, consistency, or effect. The intervention period was 2 weeks, and no wash-out period was reported	**Saliva flow rate:** Unstimulated saliva was collected using a “low force spitting” method for 10 min. Stimulated saliva was collected for 5 min using the same method with the assigned	**Saliva flow rate:** No significant effects of treatment on stimulated salivary flow rates were detected (NS)
				**Symptoms of dry mouth:** Patients were interviewed using primarily close-ended questions regarding their symptoms related to dry mouth	**Symptoms of dry mouth:** In response to a question on “What is your general opinion of the effect of the product”, the mucin chewing gum was significantly ↑ efficient in relieving dry mouth symptoms than the placebo product (**). whereas there was no difference between V6 and placebo chewing gum (NS)
Olsson et al. (1991) [38]	14 female patients with a chronic feeling of dry mouth	A single blind, randomised, cross-over design with two treatments: 1) a new chewing gum (PTC, *n* = 14), and 2) V6 chewing gum (*n* = 14)	Participants were instructed to chew the gum for 35 min The intervention period was a single session/dose with a wash-out period of at least 1-day between treatments	**Saliva flow rate:** Unstimulated saliva (baseline) was collected for 15 min. Simulated saliva secretion was measured for successive periods of 5 min (at 0, 5, 15 and 30 min)	**Saliva flow rate:** Both chewing gums significantly ↑ saliva flow rate at 7.5 after chewing was started (*)
				**Xerostomia:** Measured using oral mucosal sliding friction measurements	**Xerostomia:** Chewing the PTC gum significantly ↑ mucosal sliding friction measurements from baseline (*)
				**Self-reported saliva stimulating effect:** Measured using a 100 mm VAS	**Self-reported saliva stimulating effect:** The PTC gum had significantly ↑ ratings of saliva stimulating effect compared with V6 (*); although there was a significant treatment x period interaction with those participants who received PTC before V6 reacting differently from those who received V6 before PTC
Abelson et al. (1990) [39]	20 participants with hyposalivation secondary to salivary gland disease	A single arm study with repeated measures	Gum was chewed during 10 min stimulated saliva collection periods The intervention period was a single session	**Saliva flow rates:** Unstimulated whole mouth and parotid saliva were collected over periods of 10 min, each separated by a period of 10 min. After another 10 min period, stimulated whole mouth and parotid saliva was collected over a period of 10 min using a piece of sorbitol sweetened gum	**Saliva flow:** The mean stimulated whole mouth and parotid salivary flow rates were significantly ↑ than the unstimulated flow rates (*** and *** respectively)
Björnström et al. (1990) [40]	106 patients with low salivary flow rates and a long history of dry mouth	A multi-centre study with 8 treatments. Five were saliva stimulants: 1) Salivin, 2) V6, 3) Mucidan, 4) Ascoxal-T and 5) Nicotinamide. Three were saliva substitutes: 6) Saliment, 7) Salisynt, and 8) an ex-témpore solution	Participants were instructed to use each product according to the manufacturers’ instructions. The intervention period was 14 days with a one-week wash-out between treatments	**Saliva flow rate:** The secretion rate of paraffin-stimulated whole saliva	**Saliva flow rate:** No significant long-term effects of treatment on stimulated salivary flow rates were detected (NS)
				**Symptoms of dry mouth:** Measured using a standardised questionnaire	**Symptoms of dry mouth:** All products relieved the symptoms of dry mouth to some extent. The V6 chewing gum and Salivin lozenge were ranked as the best two products

↑ increase; ↓ decrease; * *p* < 0.05; ** *p* < 0.01; *** *p* < 0.001

In accordance with the PICOS criteria, all studies involved at least one intervention relating to the chewing of gum and a control condition (generally no chewing). Additionally, in two studies, gum chewing was also compared with sham chewing or a simple oral exercise [17, 26]. One study compared gum chewing with the sucking of a lemon-flavoured lozenge [34]. Five studies compared the effects of gum chewing with the effects of artificial saliva on associated measures of xerostomia [30, 31, 33, 34, 40].

The 25 studies included in the systematic review measured a range of different outcome variables. Twenty studies measured stimulated and/or unstimulated saliva flow rate [17–19, 21–28, 31, 32, 34–40], 17 studies measured self-reported xerostomia or symptoms of dry mouth [17–19, 21, 23–27, 29–31, 33, 34, 37, 38, 40], seven studies measured thirst [16, 20, 21, 26, 27, 29, 31], and one study measured mucosal moisture levels [22]. Of the 19 studies that measured stimulated and/or unstimulated saliva flow rate, 9 found a positive effect of gum chewing [17, 18, 22, 24, 27, 35, 36, 38, 39] and 10 did not detect any effects [19, 23, 25, 26, 28, 31, 32, 34, 37, 40]. Of the six studies included in the meta-analysis, three found a positive effect of gum chewing on unstimulated saliva flow rate [17, 18, 36], and three did not detect any effects of gum chewing on unstimulated saliva flow rate [26, 31, 34]. Of the 17 studies that measured xerostomia or symptoms of dry mouth, 12 found a positive effect of gum chewing [18, 19, 21, 23–27, 30, 31, 38, 40] and five did not detect any effects [17, 29, 33, 34, 37]. Of the seven studies that measured thirst, six found a positive effect of gum chewing [16, 20, 21, 26, 27, 31] and one did not detect any effects [29]. The single study that measured mucosal moisture found that chewing hard gum significantly increased mucosal moisture whereas chewing soft gum did not [22].

There were no results indicating that gum chewing adversely affected levels of self-reported xerostomia or symptoms of dry mouth, thirst, stimulated and/or unstimulated saliva flow rate, or mucosal moisture levels. Seven out of the 24 studies reported minor side effects associated with chewing gum use [23, 24, 29, 30, 33, 34, 36]. For the most part, the reported frequency of these events was low, and the most common symptoms were jaw pain and gastrointestinal disturbances (gas, nausea), although one study reported a high incidence of complaints of decreased appetite [29].

### Risk of bias

**Fig. 2:**
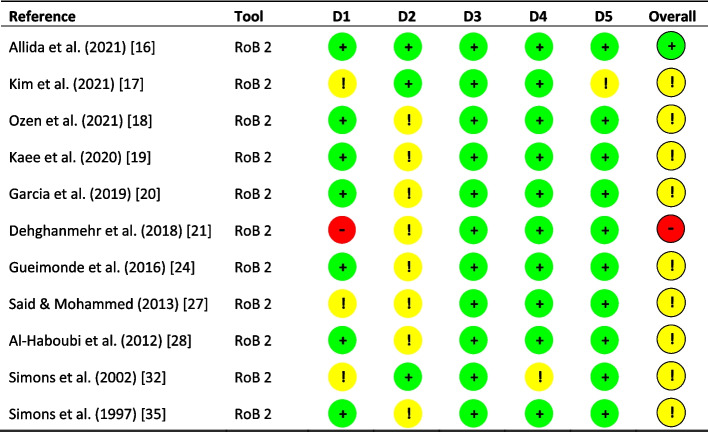
Risk of bias summary using the revised Cochrane risk of bias tool for randomised trials (RoB 2). Five domains are reported: D1 (randomisation process); D2 (deviations from the intended interventions); D3 (missing outcome data); D4 (measurement of the outcome) and D5 (selection of the reported result). ‘ + ’ low risk of bias; ‘!’ some concerns, and ‘- ‘ high risk of bias

**Fig. 3:**
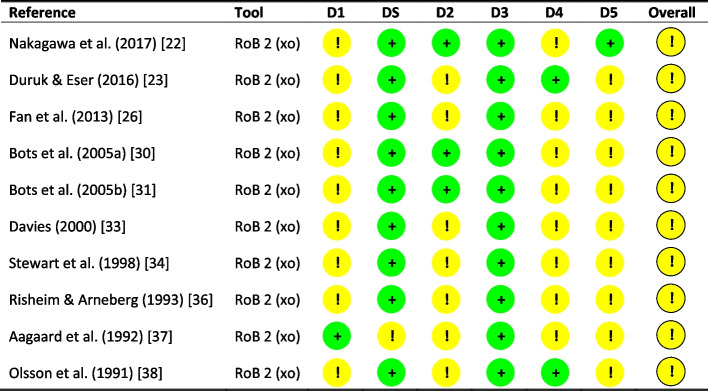
Risk of bias summary using the revised Cochrane risk of bias tool for cross-over trials (RoB 2). Six domains are reported: D1 (randomisation process); DS (bias arising from period and carryover effects); D2 (deviations from the intended interventions); D3 (missing outcome data); D4 (measurement of the outcome) and D5 (selection of the reported result). ‘ + ’ low risk of bias; ‘!’ some concerns, and ‘- ‘ high risk of bias

**Fig. 4:**
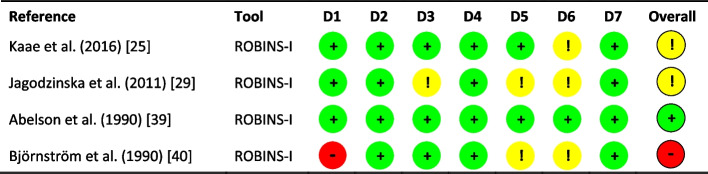
Risk of bias summary using the revised Cochrane risk of bias tool for non-randomised studies—of Interventions (ROBINS-I). Seven domains are reported: D1 (confounding); D2 (selection of participants into the study); D3 (classification of interventions); D4 (deviations from intended interventions); D5 (missing data); D6 (measurement of outcomes) and D7 (selection of the reported result). ‘ + ’ low risk of bias; ‘!’ some concerns, and ‘- ‘ high risk of bias

Eleven studies were assessed for risk of bias using the RoB 2 tool for individually-randomised, parallel-group trials [11] (Fig. 2). Of these, one had a low overall risk of bias [16]; 9 had an uncertain risk of bias [17–20, 24, 27, 28, 32, 35], and one had a high risk of bias [21]. Ten studies were assessed for risk of bias using the RoB 2 tool for cross-over trials [11] (Fig. 3). All these studies had an uncertain risk of bias [22, 23, 26, 30, 31, 33, 34, 36–38]. Four studies were assessed for risk of bias using the ROBINS-I tool [12] (Fig. 4). Of these, one had a low overall risk of bias [39]; two had an uncertain risk of bias [25, 29], and one had a high risk of bias [40]. The most prevalent sources of bias arose through incomplete or inadequate reporting on details such as randomisation, blinding, attrition and, in some cases, selective reporting.

One study did not counterbalance the order in which treatments were imposed [22], and six studies had some concerns over selection bias [17, 25, 30, 31, 36, 40] as they reported insufficient detail to fully explain how participants were selected for inclusion in the trial (Table 2). Ten studies were judged to have some concerns over the blinding of participants and personnel [17, 19, 22, 25, 27, 29–31, 36, 40]. Whilst the blinding of participants is not possible with a gum chewing intervention, study personnel should have been blinded where possible. In many cases, blinding of study personnel was not reported which resulted in some concerns over bias. Thirteen studies were judged to have some concerns over bias due absent or unreported blinding of outcome assessment [17, 19, 22, 25–27, 29–31, 34, 36, 37, 40]. Three studies were judged to have some concerns over bias due to incomplete outcome data [22, 26, 38]. In these cases it was not possible to determine whether all the participants recruited had completed the study. Levels of attrition (if present) were not reported, and degrees of freedom were not quoted to indirectly ascertain the sample sizes present in the analysis. Nine studies were judged to have some concerns over bias due to selective reporting [17, 22, 23, 25, 27, 29, 36, 37, 40]. In most cases, this risk arose through the partial reporting of either the objective (e.g. saliva flow rates) or subjective (e.g. self-reported relief from xerostomia) outcome measures. Four studies were judged to have some concerns over bias due to other sources [17, 19, 23, 25]. These sources included imbalanced groups [19], potentially confounded treatments [17], unclear statistical analysis [23] and saliva collection protocols [23, 25].

**Table 3:** Data used for the meta-analysis. Data are mean rate of unstimulated salivation (ml / min)

Study Name	Chewing duration (weeks)	Chewing			Control			Overall risk of bias
		n	mean	SD	n	mean	SD	
Kim et al. (2021) [17]	2	40	0.07	0.094	21	0.01	0.143	**Some concerns**
	5	40	0.10	0.141	21	0.03	0.066	
	8	40	0.11	0.125	21	0.05	0.077	
	11	40	0.11	0.141	21	0.06	0.154	
Ozen et al. (2021) [18]	4	28	0.13	0.050	28	0.11	0.080	**Some concerns**
	12	28	0.17	0.040	28	0.09	0.070	
Fan et al. (2013) [26]	2	11	0.24	0.240	11	0.20	0.160	**Some concerns**
Bots et al. (2005b) [31]	2	65	0.28	0.200	65	0.26	0.200	**Some concerns**
Stewart et al. (1998) [34]	2	80	0.10	0.125	80	0.07	0.134	**Some concerns**
Risheim & Arneberg (1993) [36]	2	18	0.13	0.100	18	0.09	0.007	**Some concerns**

### Effect of gum chewing on unstimulated rate of salivation

Six studies contained data suitable for inclusion in the meta-analysis (Table 3). Three of these studies were of patients undergoing dialysis [18, 26, 31], one was of an elderly population [17], one was of patients with rheumatism [36], and one was a population of patients with diagnosed xerostomia / hyposalivation [34].

Each of the studies had some overall concerns over bias. It was decided to include these studies because the primary outcome measure (salivary flow rate) was less likely to be biased by factors such as blinding of participants and personnel than subjective measures such as self-reports. However, this assumption was tested through sensitivity analysis and measures of heterogeneity.

**Fig. 5:**
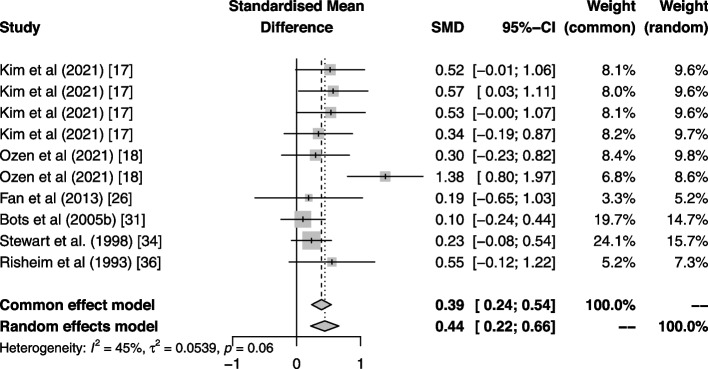
Forest plot

The meta-analysis results are presented in the forest plot in Fig. 5. There was a significant overall effect of gum on saliva flow outcomes compared to control (SMD = 0.44, 95% CI: 0.22—0.66; *p* = 0.00008; I^2^ = 46.53%). The effect is small but certain, heterogeneity is average but less than 50%.

### Heterogeneity

**Fig. 6:**
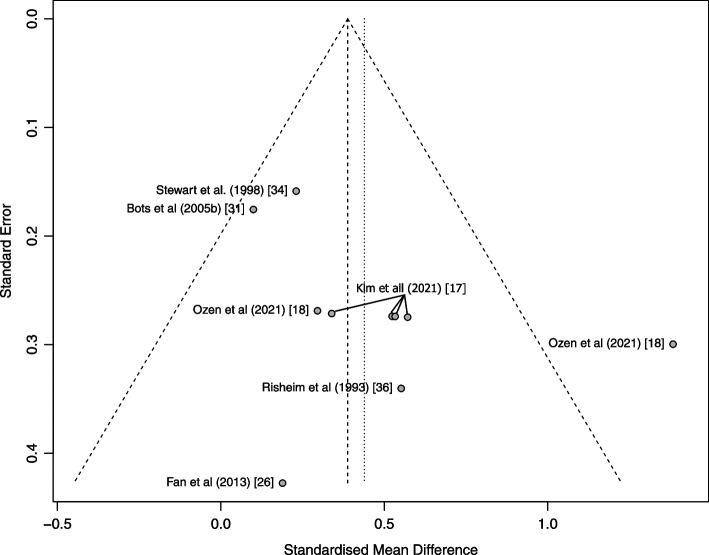
Funnel plot

Heterogeneity between the studies was assessed using graphic exploration with funnel plots in Fig. 6.

The results show that nearly all the studies are inside the funnel. One study, however, has a large SMD and lies outside the triangle as it has a large mean difference.

### Sensitivity analysis

**Fig. 7:**
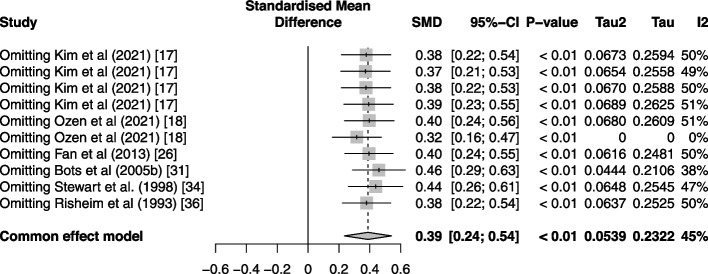
Sensitivity analysis

To assess the stability of the results and outlier analysis using a “leave one out” approach was conducted. In this approach studies were removed one by one, and the random effect model was fitted on the remaining studies. Figure 7 shows that the results remain stable for both the effect direction and magnitude.

### Effect of duration of gum chewing on unstimulated salivary flow rate

Because of the moderate heterogeneity, we used meta-regressions to investigate how the duration of the chewing intervention influenced unstimulated salivary flow rate. The results show significant decrease in heterogeneity when I^2^ is less than 1%. The results show also that the rate of salivation increased by 0.06 standard deviations per week (*p* < 0.001).

**Fig. 8:**
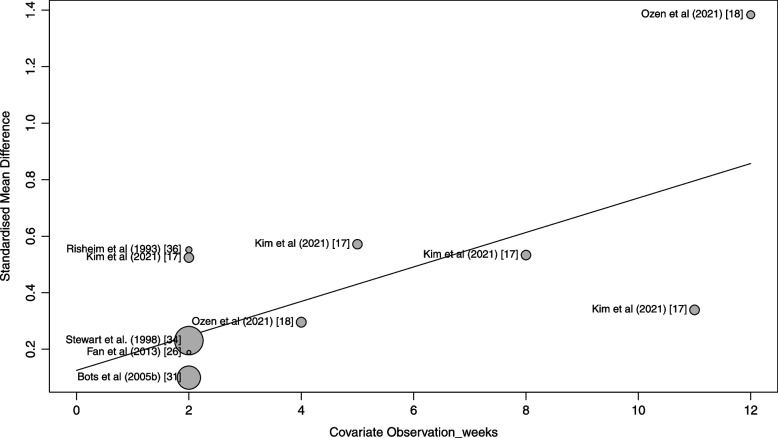
Meta-regression bubble plot

The bubble plot shows the estimated regression slope, as well as the effect size of each study (Fig. 8). To indicate the weight of a study, the bubbles have different sizes, with a greater size representing a higher weight.

## Discussion

The objective of this systematic review and meta-analysis was to investigate if gum chewing is associated with objective improvements in saliva output and subjective relief from xerostomia. Xerostomia is a condition with multiple possible aetiologies, including use of prescribed and over-the-counter medications, recreational drug use, rheumatic or autoimmune conditions, such as Sjögren’s syndrome, and radiation therapy for head and neck cancers [4]. Other associated causes include primary and secondary effects of aging [41], stress [42], and the multifactorial effects of chronic haemodialysis [43]. The physiological, neurological and cellular control of saliva secretion is complex and therefore susceptible to disruption at various levels of control. In addition, there is no consistent correlation between the subjective symptoms of xerostomia and objective measures of salivary gland function (i.e., saliva flow rates [3]). Owing to the heterogeneity of aetiological factors and the disconnect between objective and subjective measures, xerostomia is not a diagnosis or single disease entity, and certain therapeutic approaches may not be applicable to all sufferers. Both chronic haemodialysis and aging can result in salivary gland hypofunction and xerostomia due to multiple factors, including glandular fibrosis, medication use and fluid restriction [41, 43]. In contrast, Sjögren’s syndrome and radiation therapy are conditions in which the cellular secretory mechanism is irreversibly damaged and likely not responsive to stimuli such as chewing gum. However, it is apparent from several studies included in this review that stimulation of salivary flow using chewing gum has the potential to improve either subjective symptoms or objective measures of salivary output regardless of aetiology, so we elected not to constrain the breadth of the review by limiting the analysis to specific known aetiologies of xerostomia. The meta-analysis allowed assessment of the objective outcome of unstimulated whole saliva flow rate in six studies, including three conducted on kidney dialysis patients [18, 26, 31], one on subjects with self-reported xerostomia due to different medical conditions [34], one on older adults over 65 [17], and one on rheumatic patients with dry mouth [36]. Although we could not confirm that the effect of mastication or chewing gum is independent of the aetiology of the condition, we would argue the results of the meta-analysis suggests that where the effect is multifactorial (dialysis, aging), the effect of chewing was more apparent than in the studies that included patients with autoimmune conditions [34, 36] and/or post-radiation therapy [34].

### Systematic review

In accordance with the commentaries made by other reviewers [8], a large proportion of the studies reviewed included patients under dialysis [18, 21, 23, 26, 27, 29–31], or with specific conditions such as cancer [19, 25, 33], heart disease [16], or rheumatism [36]. The extent to which the aetiology of xerostomia in such studies can be compared with other studies where participants were recruited due to a history of dry mouth [20, 24, 34, 37–40] or by age [17, 22, 28, 32, 35] remains uncertain. However, chewing gum is unlikely to have an impact on xerostomia where salivary gland tissue has been ablated by radiation therapy or other catastrophic medical/surgical causes. In accordance with this proposition, the two studies of patents being treated for head, neck or oral cancers in the present review were unable detect a significant effect of gum chewing on unstimulated saliva flow rate [19, 25]. In these cases, emerging treatments such as salivary gland regeneration, repair, or replacement may be more appropriate therapies [44, 45].

Historically, a number of questionnaires have been developed to measure the presence and severity of xerostomia [46]. Initially Fox et al. (1987) defined nine questions related to xerostomia which predicted low saliva flow rates [3]. Later, the 11-item Xerostomia Inventory (XI) was proposed by Thomson et al. (1999) to develop valid multi-item method of measuring the symptoms of xerostomia which includes the wide range of xerostomia symptoms in a single quantitative measure [47]. An associated instrument was also proposed by Torres et al. (2002) which was based on 10 questions [48]. Other instruments have been developed for specific populations. For example, to measure quality of life in patients with head and neck cancer, measurements related to dry mouth / xerostomia are also included in the EORTC QLQ-H&N35 questionnaire developed by Bjordal et al. [49].

Of the studies that measured saliva flow rates, 13 studies also collected self-reported xerostomia data (e.g. xerostomia inventory [21, 26, 27, 31], EORTC QLQ-H&N35 questionnaires [19, 25], symptoms of dry mouth questionnaire [17, 24] or other questionnaire-based instruments [34, 40], visual analogue scales [18, 23, 38], and interviews [37]). There was insufficient data to conduct a meta-analysis to investigate whether increases in the rate of salivation were associated with self-reported relief from xerostomia. We had hoped to conduct a meta-analysis on self-reported data, however, the types of index used across the studies were inconsistent. It is recommended that experts align on a common instrument for future studies to allow direct comparison and meta-analysis.

Garcia et al. [8] reviewed 12 studies on the effect of chewing gum on thirst in healthy and unhealthy adults. Five of these studies found that chewing gum increased salivary flow [25, 27, 37, 38, 50]. Seven studies found that chewing gum increased xerostomia relief [26, 27, 30, 31, 33, 34, 37], and four studies found that chewing gum increased thirst reduction [26, 27, 30, 31]. Garcia et al. [8] concluded that gum chewing resulted in increased salivary flow, xerostomia relief, and thirst reduction.

### Meta-analysis

The reported effects of gum chewing on saliva flow rate across the individual studies of the systematic review were ambiguous. Nine of the 19 studies that measured stimulated and/or unstimulated saliva flow rate found a positive effect whereas 10 studies did not detect an effect (see Results section). In the absence of reported power analyses it is uncertain whether the numbers of participants were sufficient to detect an effect. However, meta-analysis can provide a more precise estimate of the effect of treatment or outcomes than any individual study contributing to the pooled analysis [51].

To the best of our knowledge, this is the first time meta-analysis has been applied to assessment of the effect of chewing gum as an intervention for dry mouth. A meta-analysis was neither included in the integrative review conducted by Garcia et al. [6], nor the Cochrane Collaboration review [7]. A recent review on the efficacy of malic acid mouth sprays on xerostomia and salivary flow rates included meta-analysis of flow rate data and, showed a significant effect of the treatment on unstimulated saliva flow rates [52] and, in the present review, our meta-analysis confirmed a small, statistically significant effect of mastication on unstimulated rate of salivation in challenged populations. An average level of heterogeneity was present in the results and the funnel plot suggested some publication bias might have been present. A meta-regression found that the duration of the intervention influences the changes in salivation rate, with longer periods of chewing being associated with higher rates of salivation (in the range 2 – 12 weeks).

Our meta-analysis could be criticised for including studies that had some concerns over the overall risk of bias. However, in many cases the potential sources of bias were a lack of reporting on issues such as blinding (which is acknowledged to be difficult / impossible in studies of gum chewing) and randomisation. To address these issues, we investigated the potential impacts of any potential biases through sensitivity analysis and by examining the levels of heterogeneity. The sensitivity analysis confirmed that no one study was leveraging the outcome and that the results remained stable for both the effect direction and magnitude.

Variation in the methodologies used in the individual studies included in the meta-analysis could have influenced the direction and effect size detected. For example, it is possible that differences in how the chewing intervention was imposed (e.g. type and flavour of gum, frequency and duration of chewing, etc.). In addition, the methodologies used to measure saliva flow rates could have influenced the outcome. A range of different chewing gums were used across the studies included in the systematic review. However, in the meta-analysis, the gum chewing interventions were more consistent. Five studies used commercially available gums sweetened with aspartame or sorbitol or xylitol [7, 32–34, 53], and one used a prototype gum produced specifically for the study (no medicinal additives) [17]. Ozen et al. (2021), Bots et al. (2005b) and Fan et al. (2013) [18, 26, 30] required participants to chew for 10 min, six times per day and when their mouth felt dry or they were thirsty. Kim et al. [17] required participants to chew for 10 min, two times per day. Stewart et al. [34] instructed participants to chew ad libitum in accordance with the manufacturer’s instructions, and Risheim & Arneberg [36] instructed participants to chew for 30 min, two times per day on days 1–4 of the intervention, and five times per day on days 5–14 of the intervention.

The systematic review found that the protocols used to measure the unstimulated rate of salivation were reasonably consistent across the studies included in the meta-analysis [17, 18, 26, 30, 34, 36]. All of the studies, with the exception of one [36], reported that participants were requested to refrain from eating and drinking pre-sampling. However, the time specified ranged from 30 min [18], to either one hour [17, 26, 30] or two hours [34] pre-sampling. In addition to restricting the consumption of food and drink, four studies also required participants to refrain from smoking [17, 26, 30, 34], and four studies required participants refrain from any oral hygiene activity such as tooth brushing [18, 26, 30, 34]. Three studies used a total saliva collection period of 5 min [17, 26, 30], whereas one used a period of 10 min [34], and another 3 min [36]. In Bots et al. [30], participants were required to rinse their mouth with tap water to alleviate thirst and xerostomia. The temperature of the water was not specified. Measurements of saliva flow rate appear to be largely unaffected by collection time, but Gill et al. (2016) found 60% of the participants had a higher saliva flow rate after rinsing with water at a temperature of 10 °C compared with water at 20 and 30 °C [54]. In many cases it was not clear at which time of day the saliva samples were collected or whether this was standardised between collection sessions. It has been found that there are significant circadian rhythms in unstimulated saliva flow rate [55]. It is possible that the small variations in the saliva collection protocols may have introduced variability into the data which, in turn, may have influenced the size of effect detected. Navazesh and Kumar (2008) described techniques for measuring unstimulated and stimulated salivary flow, including a five-minute collection time for unstimulated saliva and refraining from eating, drinking, smoking and chewing gum for one hour before collection [56]. Consistent use of these methods in future studies would facilitate easier comparisons between studies.

In a study of 191 participants (aged 18–65 years) it was found that a history of frequent gum chewing was associated with higher unstimulated salivary flow rate [57] and, in an earlier study, healthy subjects instructed to chew gum regularly for eight weeks showed increased unstimulated saliva flow rates [58]. Gueimonde et al. (2016) found twice daily gum chewing progressively increased unstimulated saliva flow rates in 52 subjects over three months, becoming significant after two months compared to baseline [24]. This makes gum chewing an attractive alternative to pharmaceutical options, either prescription or ‘over the counter’, that may be less palatable, have side-effects, or are simply not as convenient to use. There also remains the intriguing possibility that functional stimulation of the salivary glands by increased mastication over time can increase either the basal (unstimulated) saliva flow rate or stimulated flow rate, at least in subjects without physical damage to, or loss of, the acinar cells. This was suggested by the results of the meta-analysis in the present study but is also in other studies not included in this analysis. For example, Guiemonde et al. [24] reported that the mean unstimulated saliva flow rate in healthy subjects with hyposalivation increased steadily in all subjects for three months of twice-daily chewing gum use and remained at the higher level for a month following cessation of the chewing regimen. Similarly, in a study of institutionalised, frail elderly subjects who chewed gum two times a day for 12 months, Simons et al. [35] demonstrated significant incremental increases in stimulated salivary flow rates in gum chewing subjects.

### Limitations of the review and meta-analysis

A key limitation of any systematic review and meta-analysis is that of publication bias. There is a preference to publish studies that have statistically significant results despite the clinical significance of studies that do not detect significant results. In addition heterogeneity in study design, population, intervention, or outcome measures can make it challenging to draw a definitive conclusion about the effectiveness of the intervention being studied. The quality of studies included can vary, which can affect the overall quality of the review (although this can be managed to an extent through the use of risk of bias tools). Meta-analyses are typically based on summary data, making it challenging to control for confounding factors that may influence the results. Therefore, it is essential to interpret the results with caution, considering the potential sources of bias and confounding that may have affected the results.

## Conclusion

Chewing sugar-free gum is one of many options available to manage xerostomia and symptoms of dry mouth. Our review and that of other authors suggests that chewing sugar-free gum provides relief from xerostomia, and that chewing gum daily over a period of two or more weeks increases the rate of unstimulated salivation. As xerostomia negatively affects a large proportion of both the non-institutionalised older adult population (39%), and of the general population (21.3% in men and 27.3% in women), interventions involving gum chewing offer potential to improve Oral Health Quality of Life (especially in challenged populations). Sugar-free gum is low cost, readily available, safe, is not a drug, has minimal side-effects, and is generally preferred by users to other options, such as artificial salivas. However, additional work is required to unequivocally define the relationships between gum chewing, increased salivation rate and self-reported relief from xerostomia. Progress in this regard has been hampered by a lack of standardisation on the instruments used to measure self-reported xerostomia. We also recommend that future studies clearly differentiate chronic effects of chewing on salivary flow rates from the acute effects of chewing, and if possible measure both stimulated and stimulated salivary flow rates using standardised techniques as described by Navazesh and Kumar [56].

## Data Availability

All data generated or analysed during this study are included in this published article.

## References

[1] Thomson WM (2005). Issues in the epidemiological investigation of dry mouth. Gerodontology.

[2] Nederfors T, Isaksson R, Mörnstad H, Dahlöf C (1997). Prevalence of perceived symptoms of dry mouth in an adult Swedish population-relation to age, sex and pharmacotherapy. Community Dent Oral Epidemiol.

[3] Fox PC, Busch KA, Baum BJ (1987). Subjective reports of xerostomia and objective measures of salivary gland performance. J Am Dent Assoc.

[4] Guggenheimer J, Moore PA (2003). Xerostomia: etiology, recognition and treatment. J Am Dent Assoc.

[5] Locker D (2003). Dental status, xerostomia and the oral health-related quality of life of an elderly institutionalized population. Spec Care Dent.

[6] Dawes C, Macpherson LM (1992). Effects of nine different chewing-gums and lozenges on salivary flow rate and pH. Caries Res.

[7] Furness S, Worthington HV, Bryan G, Birchenough S, McMillan R. Interventions for the management of dry mouth: topical therapies. Cochrane Database Syst Rev. 2011(12):CD008934. 10.1002/14651858.CD008934.pub2 PMC13266506 22161442

[8] Garcia AKA, Fonseca LF, Furuya RK, Rabelo PD, Rossetto EG (2019). Effect of chewing gum on thirst: an integrative review. Rev Bras Enferm.

[9] Akers J, Aguiar-Ibáñez R, Baba-Akbari A (2009). Systematic reviews: CRD’s guidance for undertaking reviews in health care.

[10] Sreebny LM, Valdini A (1988). Xerostomia. Part I: Relationship to other oral symptoms and salivary gland hypofunction. Oral surgery, oral Med oral Pathol.

[11] Sterne JAC, Savović J, Page MJ, Elbers RG, Blencowe NS, Boutron I (2019). RoB 2: a revised tool for assessing risk of bias in randomised trials. BMJ.

[12] Sterne JA, Hernán MA, Reeves BC, Savović J, Berkman ND, Viswanathan M (2016). ROBINS-I: a tool for assessing risk of bias in non-randomised studies of interventions. BMJ.

[13] Rohatgi A (2021). Webplotdigitizer: Version 4.5.

[14] Follmann D, Elliott P, Suh I, Cutler J (1992). Variance imputation for overviews of clinical trials with continuous response. J Clin Epidemiol.

[15] Sawilowsky SS (2009). New effect size rules of thumb. J Mod Appl Stat methods.

[16] Allida SM, Shehab S, Inglis SC, Davidson PM, Hayward CS, Newton PJ (2021). A RandomisEd ControLled TrIal of ChEwing Gum to RelieVE Thirst in Chronic Heart Failure (RELIEVE-CHF). Hear Lung Circ.

[17] Kim H-J, Lee J-Y, Lee E-S, Jung H-J, Ahn H-J, Jung HI (2021). Simple oral exercise with chewing gum for improving oral function in older adults. Aging Clin Exp Res.

[18] Ozen N, Aydin Sayilan A, Mut D, Sayilan S, Avcioglu Z, Kulakac N (2021). The effect of chewing gum on dry mouth, interdialytic weight gain, and intradialytic symptoms: a prospective, randomized controlled trial. Hemodial Int.

[19] Kaae JK, Stenfeldt L, Hyrup B, Brink C, Eriksen JG (2020). A randomized phase III trial for alleviating radiation-induced xerostomia with chewing gum. Radiother Oncol.

[20] Garcia AKA, Furuya RK, Conchon MF, Rossetto EG, Dantas RAS, Fonseca LF (2019). Menthol chewing gum on preoperative thirst management: randomized clinical trial. Rev Lat Am Enfermagem.

[21] Dehghanmehr S, Sheikh A, Siyasari A, Karimkoshteh MH, Sheikh G, Salarzaei M (2018). Investigating the impact of sugar free gum on the thirst and dry mouth of patients undergoing hemodialysis. Int J Pharm Sci Res.

[22] Nakagawa K, Matsuo K, Takagi D, Morita Y, Ooka T, Hironaka S (2017). Effects of gum chewing exercises on saliva secretion and occlusal force in community-dwelling elderly individuals: a pilot study. Geriatr Gerontol Int.

[23] Duruk N, Eser I (2016). The null effect of chewing gum during hemodialysis on dry mouth. Clin Nurse Spec.

[24] Gueimonde L, Vesterlund S, García-Pola MJ, Gueimonde M, Söderling E, Salminen S (2016). Supplementation of xylitol-containing chewing gum with probiotics: a double blind, randomised pilot study focusing on saliva flow and saliva properties. Food Funct.

[25] Kaae JK, Stenfeldt L, Eriksen JG (2016). Xerostomia after radiotherapy for oral and oropharyngeal cancer: increasing salivary flow with tasteless sugar-free chewing gum. Front Oncol.

[26] Fan WF, Zhang Q, Luo LH, Niu JY, Gu Y (2013). Study on the clinical significance and related factors of thirst and xerostomia in maintenance hemodialysis patients. Kidney Blood Press Res.

[27] Said H, Mohammed H (2013). Effect of chewing gum on xerostomia, thirst and Interdialytic weight gain in patients on hemodialysis. Life Sci J.

[28] Al-Haboubi M, Zoitopoulos L, Beighton D, Gallagher JE (2012). The potential benefits of sugar-free chewing gum on the oral health and quality of life of older people living in the community: a randomized controlled trial. Community Dent Oral Epidemiol.

[29] Jagodzińska M, Zimmer-Nowicka J, Nowicki M (2011). Three months of regular gum chewing neither alleviates xerostomia nor reduces overhydration in chronic hemodialysis patients. J Ren Nutr.

[30] Bots CP, Brand HS, Veerman ECI, Valentijn-Benz M, Van ABM, Amerongen AVN (2005). The management of xerostomia in patients on haemodialysis: comparison of artificial saliva and chewing gum. Palliat Med.

[31] Bots CP, Brand HS, Veerman ECI, Korevaar JC, Valentijn-Benz M, Bezemer PD (2005). Chewing gum and a saliva substitute alleviate thirst and xerostomia in patients on haemodialysis. Nephrol Dial Transplant.

[32] Simons D, Brailsford SR, Kidd EAM, Beighton D (2002). The effect of medicated chewing gums on oral health in frail older people: a 1-year clinical trial. J Am Geriatr Soc.

[33] Davies AN (2000). A comparison of artificial saliva and chewing gum in the management of xerostomia in patients with advanced cancer. Palliat Med.

[34] Stewart CM, Jones AC, Bates RE, Sandow P, Pink F, Stillwell J (1998). Comparison between saliva stimulants and a saliva substitute in patients with xerostomia and hyposalivation. Spec Care Dent.

[35] Simons D, Kidd EAM, Beighton D, Jones B (1997). The effect of chlorhexidine/xylitol chewing-gum on cariogenic salivary microflora: a clinical trial in elderly patients. Caries Res.

[36] Risheim H, Arneberg P (1993). Salivary stimulation by chewing gum and lozenges in rheumatic patients with xerostomia. Eur J Oral Sci.

[37] Aagaard A, Godiksen S, Teglers PT, Schiodt M, Glenert U (1992). Comparison between new saliva stimulants in patients with dry mouth: a placebo-controlled double-blind crossover study. J oral Pathol Med.

[38] Olsson H, Spak C-J, Axéll T (1991). The effect of a chewing gum on salivary secretion, oral mucosal friction, and the feeling of dry mouth in xerostomic patients. Acta Odontol Scand.

[39] Abelson DC, Barton J, Mandel ID (1990). The effect of chewing sorbitol-sweetened gum on salivary flow and cemental plaque pH in subjects with low salivary flow. J Clin Dent.

[40] Björnström M, Axell T, Birkhed D (1990). Comparison between saliva stimulants and saliva substitutes in patients with symptoms related to dry mouth. A multi-centre study Swed Dent J.

[41] Xu F, Laguna L, Sarkar A (2019). Aging-related changes in quantity and quality of saliva: where do we stand in our understanding?. J Texture Stud.

[42] Bulthuis MS, Jan Jager DH, Brand HS (2018). Relationship among perceived stress, xerostomia, and salivary flow rate in patients visiting a saliva clinic. Clin Oral Investig.

[43] Bossola M. Xerostomia in patients on chronic hemodialysis: an update. Semin Dial. 2019;32:467–74. 10.1111/sdi.12821 31117154

[44] Hajiabbas M, D’Agostino C, Simińska-Stanny J, Tran SD, Shavandi A, Delporte C (2022). Bioengineering in salivary gland regeneration. J Biomed Sci.

[45] Rocchi C, Emmerson E (2020). Mouth-watering results: clinical need, current approaches, and future directions for salivary gland regeneration. Trends Mol Med.

[46] Villa A, Connell CL, Abati S (2015). Diagnosis and management of xerostomia and hyposalivation. Ther Clin Risk Manag.

[47] Thomson WM, Chalmers JM, Spencer AJ, Williams SM (1999). The Xerostomia Inventory: a multi-item approach to measuring dry mouth. Community Dent Health.

[48] Torres SR, Peixoto CB, Caldas DM, Silva EB, Akiti T, Nucci M (2002). Relationship between salivary flow rates and Candida counts in subjects with xerostomia. Oral Surg Oral Med Oral Pathol Oral Radiol Endodontol.

[49] Bjordal K, Ahlner-Elmqvist M, Tollesson E, Jensen AB, Razavi D, Maher EJ (1994). Development of a European Organization for Research and Treatment of Cancer (EORTC) questionnaire module to be used in quality of life assessments in head and neck cancer patients. Acta Oncol (Madr).

[50] Bots CP, Brand HS, Veerman ECI, van Amerongen BM, Amerongen AVN (2004). Preferences and saliva stimulation of eight different chewing gums. Int Dent J.

[51] Haidich A-B (2010). Meta-analysis in medical research. Hippokratia.

[52] Liu G, Qiu X, Tan X, Miao R, Tian W, Jing W. Efficacy of a 1% malic acid spray for xerostomia treatment: a systematic review and meta‐analysis. Oral Dis. 2023;29:862–72. 10.1111/odi.14116 34954846

[53] Takenouchi A, Saeki Y, Otani E, Kim M, Fushimi A, Satoh Y, et al. Effects of chewing gum base on oral hygiene and mental health: a pilot study. Bull Tokyo Dent Coll. 2021;62:7–14. 10.2209/tdcpublication.2020-0009 33583877

[54] Gill SK, Price M, Costa RJS (2016). Measurement of saliva flow rate in healthy young humans: influence of collection time and mouthrinse water temperature. Eur J Oral Sci.

[55] Dawes C (1972). Circadian rhythms in human salivary flow rate and composition. J Physiol.

[56] Navazesh M, Kumar SKS (2008). Measuring salivary flow: challenges and opportunities. J Am Dent Assoc.

[57] Wang XP, Zhong B, Chen ZK, Stewart ME, Zhang C, Zhang K (2012). History of frequent gum chewing is associated with higher unstimulated salivary flow rate and lower caries severity in healthy Chinese adults. Caries Res.

[58] Jenkins GN, Edgar WM (1989). The effect of daily gum-chewing on salivary flow rates in man. J Dent Res.

